# Associations between night eating syndrome and metabolic parameters in pregnant women

**DOI:** 10.4274/tjod.galenos.2019.77864

**Published:** 2019-07-03

**Authors:** Çiğdem Damla Deniz, Sibel Özler, Fatma Kübra Sayın, Mehmet Ali Eryılmaz

**Affiliations:** 1 University of Health Sciences, Konya Training and Research Hospital, Clinic of Medical Biochemistry Konya, Turkey; 2 University of Health Sciences, Konya Training and Research Hospital, Clinic of Perinatalogy, Konya, Turkey; 3 Necmettin Erbakan University, Faculty of Health Sciences, Department of Nutrition and Dietetics, Konya, Turkey; 4 University of Health Sciences, Konya Training and Research Hospital, Clinic General Surgery, Konya, Turkey

**Keywords:** Night eating syndrome, pregnancy, metabolic parameters in pregnancy

## Abstract

**Objective:**

In this study, we aimed to evaluate the incidence of night eating in pregnancy and the relationship between night eating scores and nutritional status, insulin resistance, and lipid profile in pregnant women.

**Materials and Methods:**

In this study, 148 pregnant women who presented to the Gynecology and Obstetrics Clinics at Konya Training and Research Hospital in Konya were divided into two groups according to their night eating scores. These two groups were compared in terms of their nutritional attitudes and metabolic parameters.

**Results:**

Comparisons of participants meeting night eating syndrome (NES) scores versus women without NES indicated that patients with NES exhibited fever hunger at breakfast time, more breakfast skipping (p<0.05) than those without NES. Also homeostatic model assessment insulin resistance, insulin, and high-density lipoprotein cholesterol parameters were significantly higher in pregnant women in the NES group (p<0.05). Also, correlations were found between higher night eating questionnaire total scores and higher HbA1c, insulin resistance, insulin, and more breakfast skipping.

**Conclusion:**

The results of this study suggest that night eating symptoms during pregnancy may increase and this is able to effect glucose metabolism.

**PRECIS:** There is an increase in night eating symptoms during pregnancy and this is able to affect glucose metabolism.

## Introduction

Night eating syndrome (NES) is a disorder characterized by evening hyperphagia and nocturnal ingestion, in addition to sleep and mood disturbances^(1)^. Evening hyperphagia is generally defined as the consumption of more than 25% of the daily calories after dinner^(2)^. In addition to these symptoms, patients may experience insomnia, a severe urge to eat at night, with morning anorexia or breakfast skipping^(3)^. For this reason, eating at night is a complex syndrome involving poor nutrition, disordered sleep patterns, and depressed mood. NES has been found associated with eating disorder (ED) attitudes and poor physical and psychosocial functioning^(3,4,5,6)^. Furthermore, people with NES have a relatively high risk of obesity, inadequate glycemic control (HbA1c values >7%), and having two or more diabetic complications^(7)^. The prevalence of NES in the adult population was reported as 1-1.5%. Although this syndrome is seen in non-obese subjects, it is more prevalent among obese individuals (6-42%), especially for attenuation therapy^(4,8,9)^.

Pregnancy can lead to an increase in night eating due to both the effect of metabolic changes and disturbed sleep patterns. Healthy nutrition and weight control knowledge during pregnancy have a great effect on the prevention of pregnancy complications. The detection of night eating problems in pregnant women is important for preventing health problems that many pregnant women and babies will be exposed to. This is the first study to highlight the relationship between NES and metabolic problems such as insulin resistance and lipid profile in pregnant women. Determining this relationship is important for preventive medicine. Thus, the aims of this study were to determine the incidence of night eating in pregnancy, and to determine the relationship between night eating scores and insulin resistance, lipid profile, and nutritional status in pregnant women.

## Materials and Methods

### Participants and procedure

A total of 148 women with singleton pregnancies were recruited from Gynecology and Obstetrics Clinics at Konya Training and Research Hospital in during their medical appointments between June 2017 and June 2018. They were at least 18 years of age. Pregnant women who were on chemotherapy or psychotropic drugs or who had diabetes mellitus were excluded from the study. All participants gave written informed consent. This study was conducted according to the guidelines laid down in the Declaration of Helsinki. Ethical approval was obtained from the Necmettin Erbakan Univercity Medical Faculty Ethics Committee Review Board (approval no: 2017/1066).

### Measures

Interviews and measurements were conducted in the clinics at recruitment and at 28-38 weeks’ gestation. Data on hunger scores in the morning and at night before sleep, breakfast skipping, and night eating scores were collected.

Hunger scores in the morning and at night during pregnancy were assessed using structured interviewer-administered questions: hunger scores were assessed by asking the question “How much do you feel hungry when you wake up?” The women were asked to choose one of 6 scores: 1) Empty; weak and light-headed. Your body is begging for food and you start to feel dizzy and nauseous. 2) Over-hungry. You feel irritable and unable to concentrate. You may even feel nauseous. 3) Hunger awakens. Slightly uncomfortable. You are just beginning to feel signs of hunger. Your body is giving you the signal that you might want to eat**. **4) Neutral/comfortable. You are more or less satisfied, but could eat a little more. Your body has enough fuel to keep it going and is physically and psychologically just starting to feel satisfied. 5) Completely satisfied. A little bit uncomfortable. You are past the point of satisfaction, yet you can still “find room” for a little more. Your body says “no” and your mind says “yes” to a few more bites. 6) Stuffed. Very uncomfortably full. You feel heavy, tired, and bloated. Higher scores reflect feeling more full.

For determining the NES, the Night Eating Questionnaire (NEQ)^(10)^ was used. Atasoy et al.,^(11)^ evaluated the reliability and validity of Turkish version of the NEQ. The NEQ is a 14-item self-report measure of NES that results in a total score with higher scores reflecting greater night eating pathologies. One item, item 13, does not assess NES pathology, but rather is used as a rule-out for sleep-related ED, and it is therefore not included in the total score calculation. A cut-off of 25 has been used to screen for NES using this measure^(10)^. In the standardization samples, the mean total scores on the NEQ were 32.4 [standard deviation (SD): 6.8] for patients with NES and 16.0 (SD: 6.3) for patients with obesity without NES who presented for bariatric surgery. The NEQ is composed of four factors (nocturnal ingestions, evening hyperphagia, morning anorexia, and sleep/mood). Each factor is represented by three items, with the exception of the nocturnal ingestions factor, which has five items. Each item has five response options, which are coded from 0 to 4, with higher scores reflecting greater impairment.

The weight and health status of the newborns were obtained from the medical charts of infants.

### Blood sample analysis

The analyses were performed in the Clinic of Biochemistry, Konya Training and Research Hospital. The plasma concentrations of the following parameters were analyzed: fasting glucose, fasting insulin, hemoglobin A1c, lipid profile, hemogram, and vitamin D. Insulin resistance, estimated by the homeostatic model assessment-method (HOMA-IR) for glucose metabolism, serum total cholesterol, low-density lipoprotein cholesterol (LDL-C), and high-density lipoprotein cholesterol (HDL-C) for lipid metabolism.

Insulin concentrations were measured using a chemiluminescent method with a Siemens immulite 2000 xPi  immunoassay analyzer (Siemens Inc.). Serum glucose and lipid concentrations were analyzed with Beckman Kits using Beckman Coulter AU 5800 biochemical analyzer (Beckman Coulter, Inc., USA). Glucose was measured using a glucose-6-phosphate dehydrogenase enzymatic assay. Triglycerides, total cholesterol, and HDL-C concentrations were determined using enzymatic colorimetry. LDL cholesterol was calculated using the Friedewald equation.

Vitamin D was analyzed using an automatic immunoassay with an Abbott Kits (Abbott Laboratories, IL 60064, USA) on architect plus i2000SR analyzer. HbA1c was determined using high-performance liquid chromatography (Trinity Biotech Premier 9210, USA).

### Statistical Analysis

All statistical analyses were performed using the SPSS version 16.0 software (IBM, Chicago, IL). The Mann-Whitney U test was used to compare the related blood parameters between the non-NES and NES group. Spearman correlations were used to determine the correlation between the night eating score with demographic, glucose metabolism, and lipid metabolism parameters. P<0.05 was considered statistically significant.

## Results

### Characteristics of the participants

A sample of 148 pregnant women (mean age 28.4 years, SD: 5.88, range 18-44 years) were included in this study. The average body mass index (BMI) was 30.66 kg/m^2^ (SD: 4.42, range 24.26-40.00 kg/m^2^).

The mean hunger score in the morning before breakfast was 2.75 (SD: 1.87), and 15% of participants scored more then 4, which means really full. The mean night hunger score was 3.61 (SD: 2.11), and 36% of participants scored more then 4, meaning that they were really full.

No significant differences in maternal characteristics were observed for age, BMI, and night eating score between women with and without NES (all p≥0.05). Morning hunger scores and breakfast skipping were significantly higher in the NES group compared with the non-NES group.

The mean score on the NEQ was 16.53 (SD: 5.88, range: 5-35) using a cut-off of 25 on the NEQ;^(12)^ 11.48% (n=17) of the participants met the criteria for NES. The means of the NES and non-NES group scores were 32.21 (SD: 5.52, range: 25-35) and 14.51 (SD: 4.78, range: 5-24), respectively.

### Comparison between NES and non-NES

Comparisons of participants meeting NES criteria versus those who did not meet the criteria (Table 1) indicated that patients with NES exhibited fever hunger at breakfast time, and more breakfast skipping than pregnant women without NES (p<0.05). Also HOMA-IR, insulin, TC, and HDL-C parameters were significantly higher in pregnant women in the NES group (p<0.05). Fetal weights of the NES group were heavier than in the non-NES group, but the difference was not significant.

### Correlations with night eating

Correlations between night eating with lipid profile, vitamin D, and glucose metabolism variables are described in Table 2. Significant small to large correlations were found between higher NEQ total scores and higher HbA1c, insulin resistance, insulin, more breakfast skipping, and feeling full before breakfast.

## Discussion

This study investigated the prevalence of night eating in the third timester of pregnancy, and also determined the relationships among night eating symptoms and relevant metabolic variables in this population.

In this study, 11.5% of participants met the screening criteria for NES, which were determined using the NEQ. As expected, the mean score in our study was higher than the mean scores of 4.5% and 4.6% in the healthy control studies. Other studies that used the NEQ as the measure of night eating in patients with diabetes found prevalence of NES as 7%, 8.4%, and 9.7%^(13,14,15)^. NES is a relatively common problem in morbidly obese individuals, and a definite diagnosis of NES was present in 10% of the obese group^(16)^. The wide range of prevalences reported across studies can be explain by the difference of assessment methods and the differences between participants in the groups. This study shows that pregnant women may have an increased tendency to night eating.

In regard to eating habits, the results indicated that increased night eating was associated with decreased morning hunger and increased breakfast skipping. This is consistent with a study of patients with diabetes that found increased breakfast skipping in patients with NES^(14)^.

According to our results in pregnant women, increased night eating scores are associated with increased insulin resistance. This is consistent with studies of patients with diabetes that found that increased NES was associated with poorer glycemic control^(7,14,16)^. Evidence of the relationship between night eating and glycemic control is mixed, possibly due to differences in symptom measurement and study samples. For example, Allison et al.,^(12) ^measured night eating symptoms using the NEQ and found no difference in HbA1c values in the NES and non-NES groups. However, in another study, Loy et al.,^(17)^ showed that increased night hunger and a reduced number of meals in the second trimester of pregnancy resulted in a decrease in fasting blood glucose and 2^nd^ hour of postprandial glucose. It has been observed that fasting for 10 hours in the control of the gestational glycemia is effective^(18)^. Gestational hyperglycemia contributes to a long-term risk for obesity in childhood,^(19)^ neonatal adiposity, and negative perinatal outcomes^(20)^. These risks occur even at blood glucose concentrations below the diagnostic limit for gestational diabetes mellitus. There is evidence that moderate glycemic recovery improves perinatal outcomes in pregnant women with mild glucose intolerance^(21)^. To date, dietary approaches to glycemic control have mostly focused on dietary quantity and quality. However, keeping the eating time range within certain limits may offer an innovative and feasible strategy to prevent gestational hyperglycemia^(22)^. Night-eating behavior can harm glycemic control, sleep patterns, and weight control in pregnant women^(23)^.

Our data confirm no association between NES and BMI in pregnant women. In a cross-sectional study of participants with diabetes, participants with NES had higher BMI and they were more likely to have unsatisfactory metabolic control. This evidence does not hold true in the obese population; Calugi et al.,^(16)^ found no differences in the prevalence of the metabolic syndrome and in its individual components between participants with and without NES. The prevalence of metabolic syndrome in the obese population is already high, so it is difficult to implicate an additional effect of NES.

EDs affect about 5-7% of women of chilbearing age. Previous studies have shown that the effect of pregnancy on ED symptoms largely remains unclear. Eating behavior in women with a history of anorexia nervosa may improve with pregnancy but appears to revert to prepregnancy concentrations^(24)^. Bulimia nevrosa symptoms during pregnancy have shown mixed results, there is evidence for worsening,^(25)^ but also for improvement of bulimia nevrosa symptoms during pregnancy^(26,27)^. A study of Crow et al.,^(28)^ showed that pregnancy had an improving effect on binge eating and purging in women who had had eating desorders before pregnancy. In another study, the proportion of women meeting criteria for binge ED was found to increase during pregnancy.

### Study Limitations

The literature is limited to EDs such as anorexia, bulimia, and binge eating. In our study, it is seen that NES does not tend to improve in pregnancy in contrast to other EDs.

## Conclusion

To our knowledge, no studies of night eating symptoms during pregnancy have been conducted. The results of this study suggest that night eating symptoms during pregnancy may increase and this is able to affect glucose metabolism. Our findings can be evaluated as follows: pregnancy can lead to changes in eating attitudes and behaviors, even in women without EDs. Future research in this area should use larger samples to examine the possibility of metabolic effects of NE symptoms during pregnancy.

## Figures and Tables

**Table 1:**
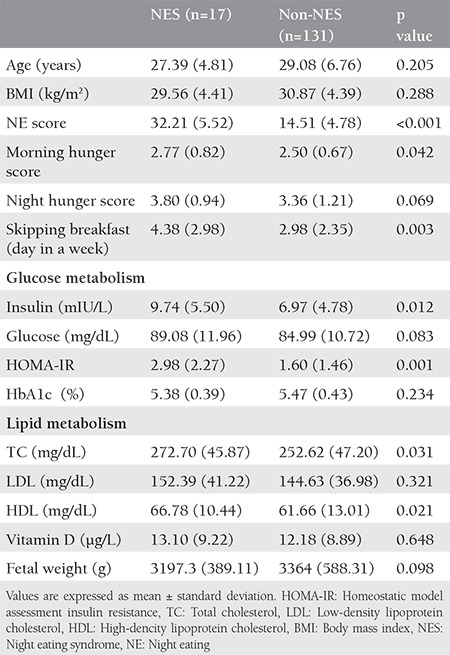
Comparison between pregnants with and without NES

**Table 2:**
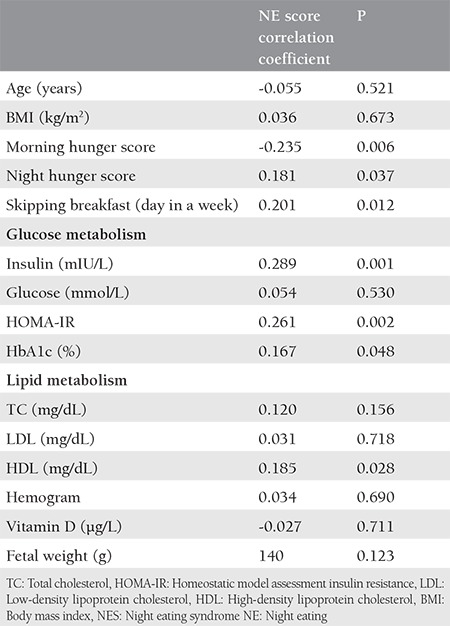
Correlations with NES
